# Local metastatic expansion versus secondary intra-organ dissemination: two causes of neurological death explained by fundamentally different metastatic colonization patterns

**DOI:** 10.1186/s12943-026-02574-0

**Published:** 2026-01-24

**Authors:** Dorde Komljenovic, Tobias Bäuerle, Jessica Alves-de-Lima, Laura Trigueros, Cara Dietz, Zoltan Winter, Tommaso Araceli, Quirin Strotzer, Christina Wendl, Matthias Brendel, Martin A. Proescholdt, Patrick N. Harter, Katja Evert, Tobias Pukrop, Raquel Blazquez

**Affiliations:** 1 Institute of Radiology, University Hospital Erlangen, Friedrich-Alexander-Universität Erlangen-Nürnberg, Erlangen, Germany; 2 Preclinical Imaging Platform Erlangen (PIPE), Department of Radiology, University Hospital Erlangen, Friedrich-Alexander-Universität Erlangen-Nürnberg, Erlangen, Germany; 3 Department of Diagnostic and Interventional Radiology, University Mainz Medical Center, Mainz, Germany; 4 Department of Internal Medicine III, Hematology and Medical Oncology, University Hospital Regensburg, Regensburg, Germany; 5 Department of Neurosurgery, University Hospital Regensburg, Regensburg, Germany; 6 Department of Radiology, University Hospital Regensburg, Regensburg, Germany; 7 Department of Nuclear Medicine, University Hospital, LMU Munich, Munich, Germany; 8 German Center for Neurodegenerative Diseases (DZNE), Munich, Germany; 9 German Cancer Consortium (DKTK), Partner Site Munich, German Cancer Research Center (DKFZ), Heidelberg, Germany; 10 Munich Cluster for Systems Neurology (SyNergy), University of Munich, Munich, Germany; 11 Bavarian Cancer Research Center (BZKF), Erlangen, Germany; 12 Center for Neuropathology and Prion Research, Faculty of Medicine, Ludwig- Maximilians-University Munich, Munich, Germany; 13 Bavarian Cancer Research Center (BZKF), Munich, Germany; 14 Institute of Pathology, University of Regensburg, Regensburg, Germany; 15 Bavarian Cancer Research Center (BZKF), Regensburg, Germany; 16 Center for Translational Oncology (CTO), University Hospital Regensburg, Regensburg, Germany; 17 Fraunhofer Institute for Toxicology and Experimental Medicine, ITEM-R, Regensburg, Germany

**Keywords:** Brain metastasis, Cause of death, Histological growth pattern, Infiltration, Local metastatic expansion, Meningeal metastasis, MMPI, Neurological decline, Recolonization, Secondary dissemination

## Abstract

**Background:**

Neurological failure contributes to 15–50% of deaths in patients with brain metastases, yet the underlying mechanisms remain poorly understood. Clinical causes range from local compression to meningeal metastasis. In this context, a link between infiltrative histopathological growth patterns (HGPs) and meningeal metastasis was recently described and prompted this reverse translation study.

**Methods:**

We conducted a retrospective postmortem histological assessment and a prospective MRI-based proof-of-concept study to explore neurological decline mechanisms in two experimental brain metastasis models with different HGPs: (i) the non-infiltrative TUBO model, characterized by well-defined tumor borders and a multilayered astrocytic capsule; and (ii) the infiltrative E0771-LG model, exhibiting diffuse infiltration and widespread astrogliosis.

**Results:**

In the TUBO model, neurological death resulted from local metastatic expansion compressing vital structures, while the E0771-LG model caused mortality mainly through widespread secondary dissemination. We provide the first direct evidence of contralateral recolonization by secondary metastasis-initiating cells (secMICs), and highlight the high efficiency of secondary spread. Additionally, we show that secMICs exploit distinct anatomical structures to reach distant brain regions, bypassing classical vascular dissemination routes. Notably, the HGP and its associated features are intrinsic to tumor cells and are established early during metastatic colonization.

**Conclusions:**

This study identifies the HGP as a potential surrogate for predicting the underlying cause of organ failure in brain metastases. Additionally, it highlights the significant role of secondary dissemination and recolonization in brain metastasis, processes that have been largely overlooked in clinical practice. These findings address a critical knowledge gap and may inform future treatment strategies.

**Supplementary Information:**

The online version contains supplementary material available at 10.1186/s12943-026-02574-0.

## Background

The steadily rising incidence of brain metastases is contributing to an increasing number of cancer-related deaths due to central nervous system (CNS) failure [1]. In some cancer types, CNS failure has already become the leading cause of cancer-related mortality. This is particularly true for patients with non-small cell lung cancer (NSCLC) harboring EGFR mutations [2, 3], HER2-positive breast cancer [4], and malignant melanoma [5]. Despite the high frequency of neurological death in patients with brain metastases, the pathophysiological mechanisms of CNS failure remain poorly understood [6].

The Monro-Kellie doctrine has significantly contributed to our understanding of the potential mechanisms underlying neurological death. Formulated in the 19th century, the doctrine states that the total volume of brain tissue, blood, and cerebrospinal fluid (CSF) within the non-expandable cranial vault must remain constant to preserve stable intracranial pressure (ICP) [7]. In the context of brain metastases, any increase in intracranial volume—such as that caused by tumor growth—must be offset by a reduction in one of the other components. If this compensatory mechanism fails, it results in a rapid rise in ICP, which can ultimately lead to organ failure. Several mechanisms contribute to a threatening increase in ICP in brain metastasis. These include direct tumor expansion, the development of perimetastatic edema, and vascular erosion leading to intracranial hemorrhage [6]. Additionally, CNS failure may result from increased CSF volume due to impaired circulation or absorption. Mechanical obstruction (e.g. congestion at the cerebral aqueduct of Sylvius) or resorptive dysfunction associated with meningeal metastases are common examples. These scenarios highlight at least five distinct pathophysiological processes that can independently or collectively lead to CNS failure and subsequent neurological death in the context of brain metastases.

Even though these pathophysiological processes leading to neurological death are well known to happen in patients with brain metastasis, current experimental models often fail to systematically investigate the underlying causes of organ dysfunction. Most in vivo metastasis studies prioritize primary endpoints such as weight loss, behavioral or neurological abnormalities, or other predefined clinical termination criteria. These endpoints are typically used to assess overall survival or metastatic progression, while the specific mechanisms driving neurological decline are frequently overlooked. Tumor volume and the number of metastatic lesions are commonly analyzed as secondary endpoints, rather than being directly linked to functional decline or CNS failure. Furthermore, even when varying treatment responses are observed across different models, postmortem analyses rarely include autopsy-based assessments of the specific causes of CNS failure—despite the fact that the relevant tissue is readily available. This gap underscores the need for more detailed and mechanistically focused studies to better understand the ultimate causes leading to neurological death in brain metastasis. A similar lack of analytical depth exists in clinical practice. Comprehensive postmortem investigations are rare [8], limiting our ability to identify the precise determinants of death in patients with advanced disease. For instance, in the United States, the autopsy rate for cancer patients has fallen below 1% [9], making it exceedingly difficult to systematically study the terminal pathophysiological events associated with organ death.

We previously reported that the histological growth pattern (HGP) of brain metastases at the interface with surrounding brain tissue—referred to as the macro-metastasis–brain parenchyma interface (MMPI_brain_)—may influence disease progression and ultimately indicate the potential pathophysiological reason of the cause of death [6]. Brain metastases exhibit distinct growth patterns at the MMPI_brain_: they may either displace adjacent brain tissue (non-infiltrative HGP) or infiltrate it (infiltrative HGP). Infiltrative HGPs can be further classified into epithelial and diffuse subtypes, with the diffuse type typically associated with mesenchymal-like features and deeper parenchymal infiltration [10, 11]. Recent evidence suggests a possible association between the infiltrative behavior of brain metastases and the development of meningeal metastases [12, 13], but this correlation remains insufficiently explored. Therefore, additional research is needed to investigate the role of the HGP of brain metastases as a potential morphological marker for predicting the neurological cause of death.

Moreover, one of the most fundamental questions in metastasis biology—whether metastases can themselves give rise to new metastases—remains unresolved. The prevailing paradigm holds that only the primary tumor serves as the source of metastasis-initiating cells (primary MICs or primMICs), while metastatic lesions lack the capacity for further dissemination [14]. This view is largely based on the assumption that metastasis-initiating cells (MICs) originating from secondary sites (secondary MICs or secMICs) do not have sufficient time or opportunity to complete the full metastatic cascade. However, if secMICs were able to disseminate early during the colonization of a secondary site, they might circumvent certain steps in the cascade and potentially seed new lesions more efficiently [15]. This concept of secondary dissemination remains largely unexplored, especially in the context of brain metastases [6]. It is also unclear whether all metastases possess this capacity, or if it depends on specific biological or histological traits. If such fundamental differences exist, they could significantly influence disease progression and potentially determine the pathophysiological mechanisms leading to neurological death.

Building on these insights, we hypothesize that the HGP at the MMPI_brain_ could be a potential predictor for the ultimate cause of death in brain metastases patients. To test this hypothesis, we employ two breast cancer brain metastasis models—one with a diffuse infiltrative HGP exhibiting early secondary dissemination and another showing a non-infiltrative HGP and expansive growth without significant secondary spread. Through comparative analysis, we aim to elucidate how the architecture of the tumor–parenchyma interface influences metastatic behavior and contributes to the mechanisms leading to neurological death.

## Methods

### Cell culture

E0771-LG and TUBO cells were kindly provided by Prof. J. Pollard (London, United Kindom) and Prof. C. Klein (Regensburg, Germany), respectively, and grown in Dulbecco’s Modified Eagle’s Medium (DMEM, Sigma-Aldrich, Taufkirchen, Germany) with 10% fetal calf serum (FCS, Bio&SELL, Feucht, Germany) at 37 °C in a humidified 5% CO_2_ atmosphere. Cells were routinely tested for mycoplasma contamination and authenticated based on morphological assessment using light microscopy.

For injection experiments, tumor cells were trypsinized during the exponential growth phase and resuspended at a density of 10^3^ cells in 3 µL Matrigel-DMEM supplemented with 10% FCS (2:1). The cell suspension was kept on ice until inoculation in the mouse, for a maximum of two hours.

### Experimental models of brain metastasis

Female BALB/c and C57BL/6 mice (10–12 weeks old) were obtained from Charles River Laboratories (Sulzfeld, Germany) and housed under standard conditions in the Central Animal Facilities (ZTL) of the University of Regensburg (Germany).

Stereotactic intracerebral injections were performed under anesthesia, as previously described [16–19]. Murine tumor cells were injected into the basal ganglia (caudate putamen/striatum) of the right brain hemisphere in syngeneic, immunocompetent mice. E0771-LG cells were injected in C57BL/6 mice, while TUBO cells in BALB/c mice. Control animals received equivalent injections of Matrigel–DMEM 10% FCS without tumor cells.

Unless stated otherwise, mice were monitored until the onset of neurological symptoms, at which point they were euthanized. Neurological decline was assessed in a blinded manner using the Hanging Wire test, as previously described [19]. No randomization was applied. Following euthanasia, brains were harvested, sectioned, and prepared for histological analysis. Slides were digitized and archived in a digital slide library for further evaluation.

For magnetic resonance imaging (MRI) analysis, animals were transferred to the Preclinical Imaging Platform Erlangen (PIPE, Erlangen, Germany) three days after tumor cell implantation.

### Calculation of colonization index

The colonization index (CI) is a mathematical equation enabling the comparison of the colonization capacity among different tumor cell lines in the brain, which includes the rate of mice that develop metastasis within 140 days (successful colonization, %), the number of injected cells (N), and the time to onset of neurological symptoms (days).

$$\:CI=\:\frac{successfully\:colonized\:animals\:\left(\%\right)}{injected\:cells\:\left(N\right)\:x\:time\:to\:neurol.\:symptoms\:\left(days\right)\:}\:x\:100$$

### Immunohistochemistry (IHC) of murine BM sections

Tissue sections were deparaffinized, stained with H&E or pretreated for IHC using standard techniques. An anti-E-cadherin (Ecad) or anti-Vimentin (Vim) antibody was used to detect tumor cells in TUBO-BM and E0771-LG-BM, respectively. Further, adjacent sections were stained using an anti-Glial Fibrillary Acidic Protein (Gfap) antibody to label the adjacent brain tissue. A double staining with anti-CD31 and anti-Vim antibodies was performed to simultaneously visualize blood vessels and E0771-LG tumor cells, respectively.

### In vivo magnetic resonance imaging in BM mouse models

Animals were imaged on days 14, 20 and 22 after tumor inoculation using a preclinical 7 T MRI scanner (BioSpec, Bruker BioSpin, Ettlingen, Germany) equipped with a mouse brain coil RF SUC 300 1 H M.BR. QSN RO AD (Bruker, Ettlingen, Germany). For all examinations, animals were anaesthetized by inhalation of a mixture of oxygen (0.5 l/min) and isoflurane (1.5 vol %). The respiratory rate was monitored and maintained at 60 breaths/min to ensure a stable anesthetic plane throughout the procedure. A gadolinium-based contrast agent (0.15 mmol/kg Gadovist (Gadobutrol), Bayer Vital GmbH, Leverkusen, Germany) was administered via a tail vein catheter.

Following imaging sequences were used: horizontal T2-weighted RARE (TR 2200 ms, TE 36ms, matrix 361 × 298, resolution 0.050 × 0.050 mm, 4 averages, scan time 5:25 min) and coronal T1-weigted RARE before and after the administration of the contrast agent (TR 750 ms, TE 6.50 ms, matrix 265 × 281, resolution 0.060 × 0.060 mm, 3 averages, scan time 5:15 min). Plane nomenclature was assigned according to [20].

### Calculation of tumor volume by MRI

Tumor volumes [mm^3^] were assessed using OsiriX PRO software (version 2.08.006) by means of manual segmentation primarily on horizontal T2-weighted images in close correlation with contrast-enhanced T1-weighted images especially in case of poorly demarcated tumors. Three-dimensional reconstructions of control and tumor-bearing mouse brains were obtained by using a segmentation tool 3D Slicer [21].

### Statistical analysis

Unless indicated otherwise, all values were expressed as means ± standard deviation (SD). Statistical differences were analyzed by Student’s t-test, one-way ANOVA, Fisher´s exact test or log-rank test using GraphPad Prism software version 10 (GraphPad, San Diego, CA). A p-value of < 0.05 was considered statistically significant.

## Results

### Retrospective histological assessment of mechanisms contributing to the cause of death in experimental models of brain metastasis

**Fig. 1:**
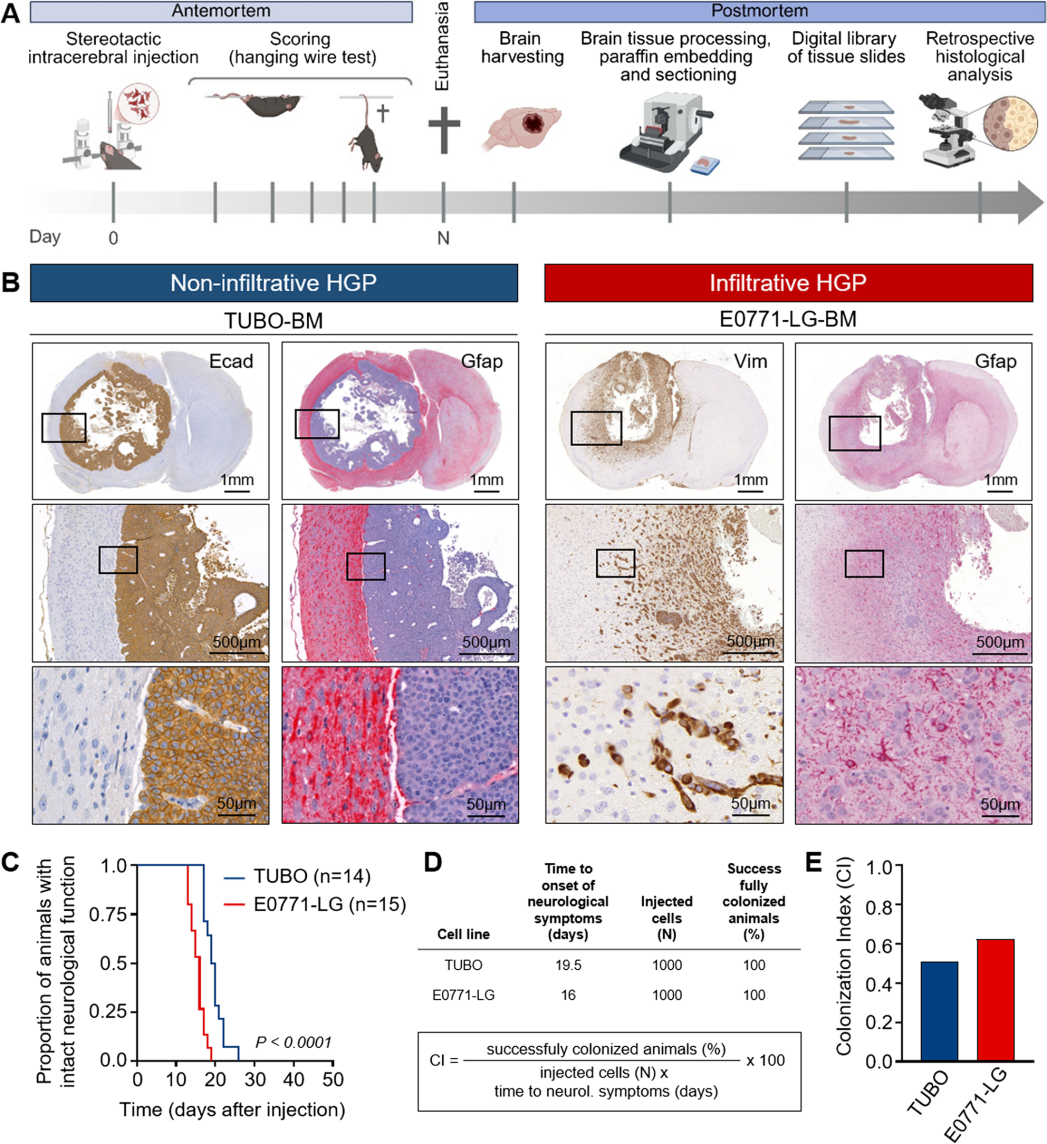
Experimental models of brain metastasis with infiltrative vs. non-infiltrative HGPs.** A** Schematic overview of the experimental workflow. The diagram illustrates the animal models used (antemortem phase), including tumor cell injection and scoring, as well as the postmortem procedure for brain tissue collection, processing, and retrospective histological analysis. Image created with Biorender.com. **B** Representative pictures of brain metastasis (BM) with non-infiltrative (TUBO-BM) and infiltrative (E0771-LG-BM) HGPs. Tumor cells in tissue sections of TUBO-BM and E0771-LG-BM were stained with E-cadherin (Ecad) or vimentin (Vim), respectively. The brain tissue was visualized with anti-Gfap. **C** Kaplan-Meier curve depicting the proportion of mice retaining intact neurological function in the TUBO-BM (blue) and E0771-LG-BM (red) models. Statistical analysis was performed with the Log-rank test (*P* < 0.0001). **D** Summary of parameters used to calculate the Colonization Index (CI). The table above lists all variables from both models incorporated into the CI formula shown below. **E** Colonization Index (CI) of TUBO-BM (blue) and E0771-LG-BM (red)

We aimed to identify histopathological factors potentially involved in the neurological death in brain metastasis. To do this, we conducted a retrospective evaluation of archived tissue slides from previous experiments using established brain metastasis models, drawing from our digital slide library (Fig. 1A). For comparative analysis, we selected digital tissue slides from the two breast cancer brain metastasis (BCBM) models that exhibit the most pronounced differences in their histological growth patterns (HGPs) at the MMPI_brain_. Specifically, we compared the HER2-driven TUBO brain metastasis (BM) model, which displays a purely non-infiltrative HGP characterized by well-demarcated tumor borders and a localized multilayered astrocytic capsule (Fig. 1B, left), with the triple-negative E0771-LG-BM model, which shows a diffuse infiltrative HGP. In contrast to the non-infiltrative TUBO-BM model, metastatic E0771-LG cells infiltrate beyond the activated astrocytes, penetrating deeply into the brain parenchyma and inducing widespread astrogliosis (Fig. 1B, right). The E0771-LG-BM model was associated with a statistically significant, albeit not clinically relevant, reduction in survival, as reflected by the proportion of animals retaining intact neurological function, compared with the TUBO-BM model (mean OS = 16 vs. 19.5 days, respectively, Fig. 1C) and displayed, consequently, a higher colonization index (Fig. 1D-E).

**Fig. 2:**
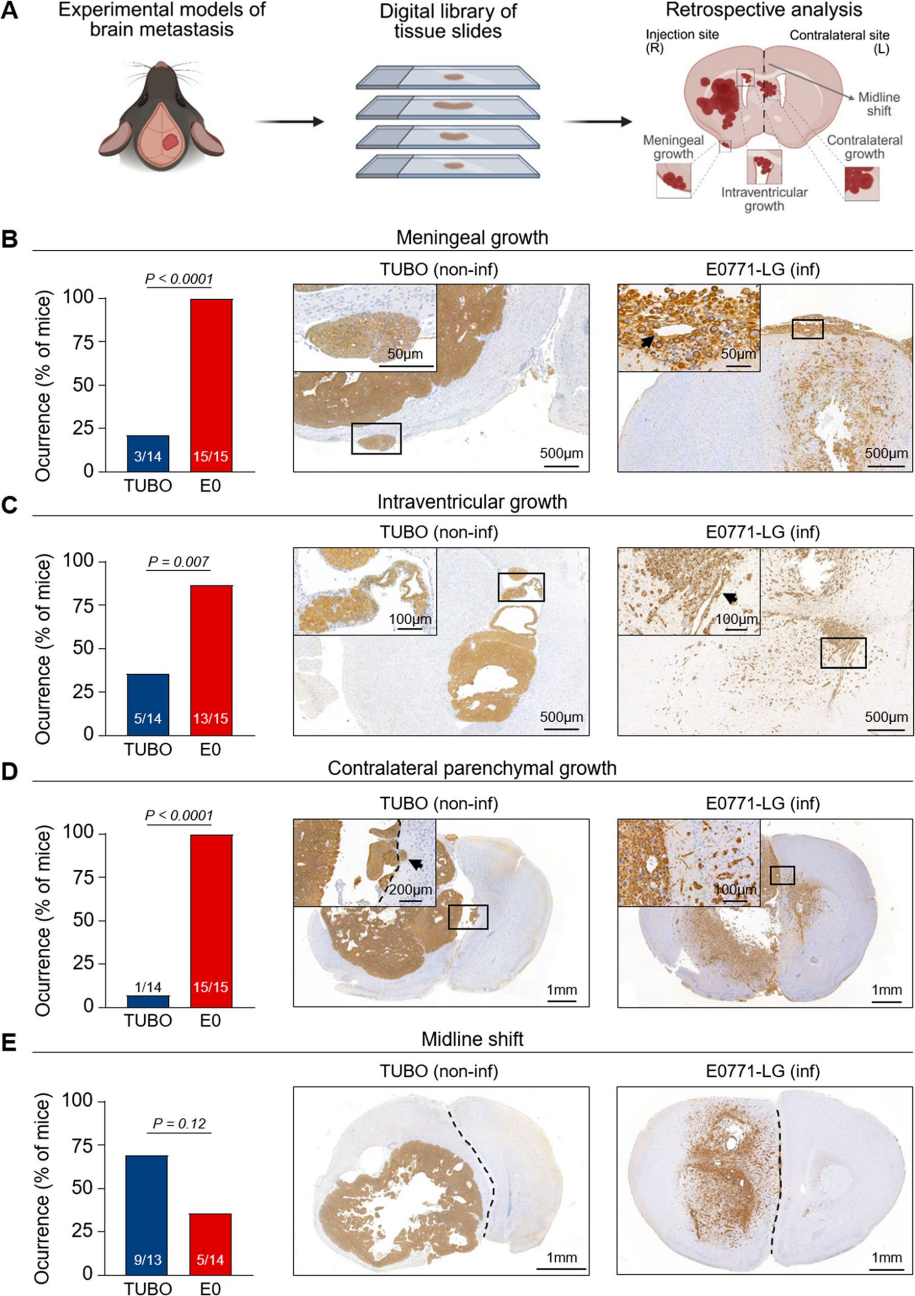
Retrospective histological evaluation of tumor growth in experimental models of brain metastasis with infiltrative vs. non-infiltrative HGPs. **A** Schematic overview of the experimental workflow. Histological factors associated with neurological decline were retrospectively analyzed in tissue slides obtained from the digital library. Image created with Biorender.com. **B**-**E** Quantification of meningeal growth (**B**), intraventricular growth (**C**), contralateral parenchymal growth (**D**) and midline shift (**E**) occurrence in TUBO-BM and E0771-LG-BM. Statistical analysis was performed with the Fisher´s exact test. Tumor cells in tissue sections of TUBO-BM and E0771-LG-BM were stained with E-cadherin (Ecad) or vimentin (Vim), respectively. Representative pictures are shown

We retrospectively analyzed tissue slides from our digital library to identify potential histopathological features contributing to neurological death in both models, including meningeal involvement, intraventricular spread, contralateral parenchymal growth, and midline shift (Fig. 2A). To minimize bias and enable direct model comparison, tumor cells were stereotactically injected at the same brain location, and brain sections were cut and stained systematically, ensuring consistent evaluation across regions [19]. Slides were selected from the digital archive based on three criteria: (1) presence of metastatic lesions (2) complete sections including both hemispheres, and (3) evaluable HGP. One representative slide per animal meeting all criteria was chosen for detailed analysis.

This retrospective histopathological evaluation revealed that meningeal involvement was more frequent in E0771-LG-BM compared to TUBO-BM (100% vs. 21.4%, respectively; *p* < 0.0001; Fig. 2B). Similarly, the presence of tumor cells within the ventricles was observed more often in the E0771-LG-BM model than in the TUBO-BM model (86.7% vs. 35.7%, respectively; *p* = 0.007; Fig. 2C). Furthermore, all E0771-LG-BM specimens showed metastatic growth in the contralateral (left) hemisphere, whereas TUBO-BM lesions remained largely confined to the injection site in the right hemisphere, with only one exception (100% vs. 7.1%, respectively; *p* < 0.0001; Fig. 2D). Interestingly, secondary metastases (M2), whether parenchymal or meningeal, maintained the same HGP as the initial metastasis (M1) in both models. E0771-LG-BM typically showed single-cell infiltration, often perivascular, while TUBO-BM exhibited a compact epithelial growth (Suppl. Figure 1). A notable finding was that midline shift (an indicator of increased intracranial pressure) was slightly more frequently observed in the non-infiltrative TUBO-BM model than in the infiltrative E0771-LG-BM (69.2% vs. 35.7%, respectively; *p* = 0.12; Fig. 2E). Although this difference did not reach statistical significance, TUBO-BM exhibited a significantly enlarged injected hemisphere compared to controls, whereas E0771-LG-BM did not (Suppl. Figure 2). Altogether, these data suggest that local metastatic expansion with compression of vital brain structures (including the brainstem) caused by the expansion of a solitary metastasis is the primary contributor to neurological decline in the non-infiltrative model.

### Prospective MRI visualization of metastatic progression in experimental brain metastasis models

Our retrospective postmortem histological analysis revealed fundamental differences in the histopathological mechanisms driving metastatic progression, and ultimately the cause of death, between infiltrative and non-infiltrative brain metastasis models. In the non-infiltrative TUBO-BM model, disease progression was primarily driven by the expansion of the first lesion (M1), leading to increased hemisphere size and midline shift (Fig. 2 and Suppl. Figure 2), consistent with the Monro-Kellie doctrine [22]. In contrast, the infiltrative E0771-LG-BM model showed midline shift in fewer animals than the non-infiltrative model (Fig. 2E), and this shift did not lead to a significant increase in hemisphere size compared with controls (Suppl. Figure 2), suggesting only a marginal contribution to neurological decline. Instead, this model caused mortality through extensive secondary dissemination, including infiltration of the meninges, ventricles, and contralateral hemisphere (Fig. 2).

Astrocytic responses also differed markedly between the models. In TUBO-BM, the metastatic lesion appeared to be contained locally by an astrocyte border, made of densely packed, overlapping, and multilayered astrocytic protrusions (glial scar-forming astrocytes). In contrast, E0771-LG-BM induced a diffuse gliosis that was more loosely organized and spatially disconnected from the MMPI_brain_ (Fig. 1B).

**Fig. 3:**
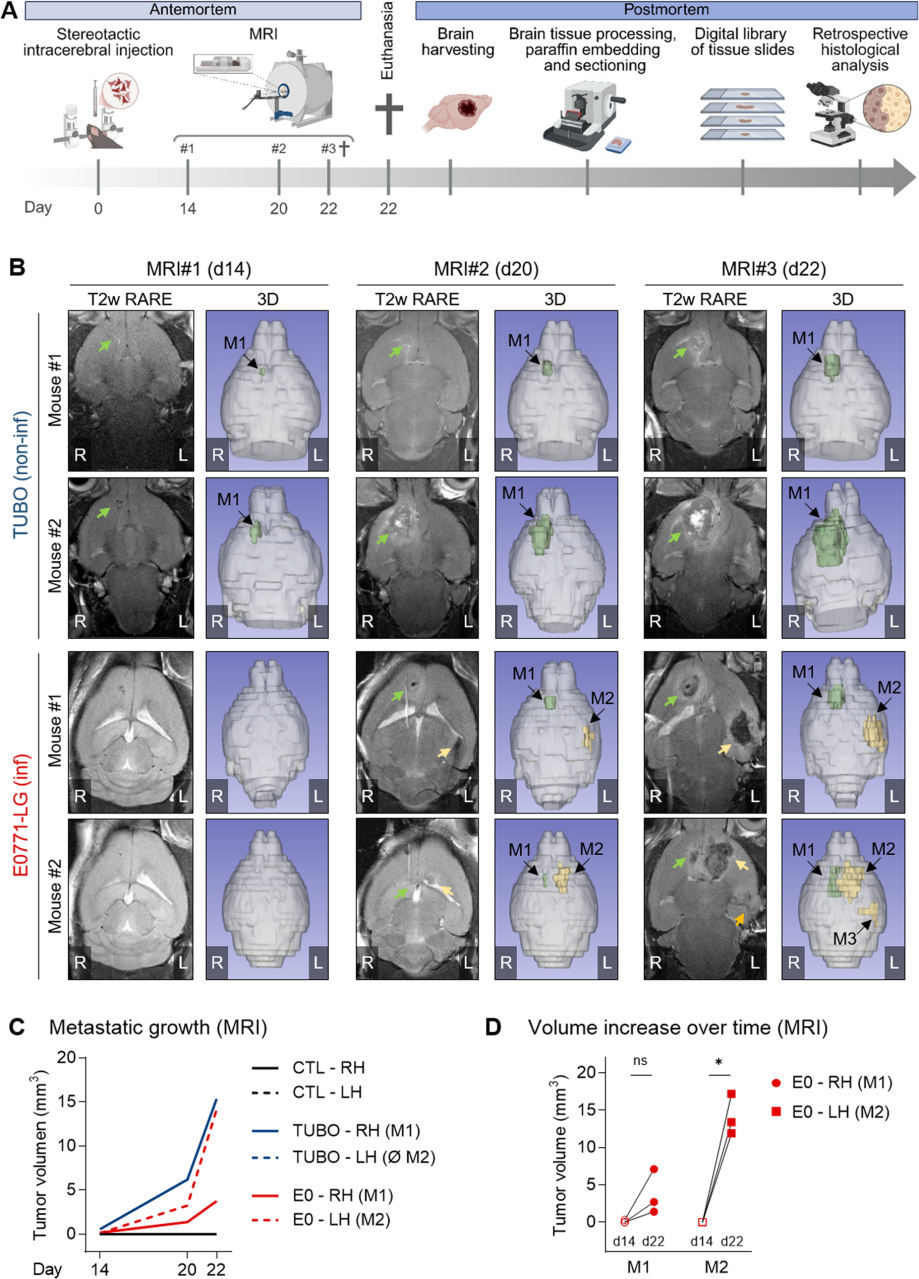
Prospective MRI evaluation of growth dynamics in experimental models of brain metastasis with infiltrative vs. non-infiltrative HGPs. **A** Schematic overview of the experimental workflow. The diagram illustrates the MRI monitoring conducted during the antemortem phase following stereotactic intracerebral injection, as well as the postmortem workflow for brain tissue collection, processing, and histological analysis. Image created with Biorender.com. **B** Horizontal T2-weighted MR images of mouse brains and 3D reconstruction pictures of TUBO-BM and E0771-LG-BM at day 14 (MRI#1), 20 (MRI#2) and 22 (MRI#3) after tumor cell inoculation. Two mice per experimental model are shown. The arrows indicate the metastatic lesions (M). Primary (M1) and subsequent lesions (M2, M3) are depicted in green or yellow, respectively, in the 3D pictures. **C** Quantification of the tumor volume (mm^3^) in the right (RH, solid line) or left hemisphere (LH, dashed line) of control (CTL) mice (black), TUBO-BM (blue) and E0771-LG-BM (red) in MRI T1w RARE scans. Mean values are shown (CTL *n* = 3, TUBO-BM *n* = 2, E0771-LG *n* = 3). **D** Volume increase over time in E0771-LG-BM M1 (red dots), and M2 (red squares) at day 14 (filled symbols) and 22 (empty symbols) after tumor cell inoculation. Paired t-test (day 14 vs. 22; *n* = 3; M1 = ns; M2 *P* = 0.02)

To visualize these differences in vivo and assess the growth dynamics of these models in detail, we next conducted a prospective MRI-based proof-of-concept study. A small group of experimental animals (*n* = 2–3 per model) were stereotactically injected with either ECM alone (control), TUBO or E0771-LG tumor cells, and metastatic progression was monitored using dedicated small-animal MRI on days 14, 20, and 22 post-injection. At the study endpoint (day 22), brains were harvested for postmortem histological analysis (Fig. 3A).

In control mice, no metastatic lesions were observed at any time point, with MRI scans showing only the inoculation canal (Suppl. Figure 3 A-B). In the TUBO-BM model, a detectable metastatic lesion (M1) appeared in the right (ipsilateral) hemisphere by day 14 and expanded exponentially through the end of the study (Fig. 3B, upper panel). This growing lesion led to a noticeable midline shift in all mice from day 20 onward (Suppl. Figure 3B, middle panel), consistent with findings from the retrospective histological analysis (Fig. 2E). These results provide evidence of mass effect with midline shift and downward displacement of brain structures in TUBO-BM and reinforce the notion that progressive expansion of a single, localized metastasis is the pathophysiological driver of neurological death in the non-infiltrative model.

In contrast, the initial lesion (M1) in the E0771-LG-BM model in the right hemisphere was first detected on day 20 and was immediately accompanied by a second lesion (M2) in the left (contralateral) hemisphere. These secondary lesions were typically located near the falx cerebri or adjacent to the lateral ventricle. By day 22, a third lesion (M3) was observed in one of three mice (Fig. 3B, lower panel). Notably, only a minor midline shift was seen in a single E0771-LG-BM mouse (Suppl. Figure 3B, lower panel), corroborating previous histological observations (Fig. 2E).

MRI-based tumor volume analysis confirmed exponential tumor growth in the right hemisphere (RH) of TUBO-BM mice and in both hemispheres (RH = M1 and LH = M2) in the E0771-LG-BM model (Fig. 3C). Strikingly, the M2 in the E0771-LG-BM model exhibited a growth rate 3.5 times faster than that of the M1 (Fig. 3D), suggesting highly efficient recolonization by secondary metastasis-initiating cells (secMICs).

In summary, this exploratory prospective MRI study, coupled with postmortem histological validation, reveals that although both models produce clinical symptoms within a similar timeframe, the underlying pathophysiological mechanisms leading to CNS failure and death are fundamentally distinct. While the non-infiltrative TUBO-BM model causes death through localized mass expansion with compression of vital brain structures (including the brainstem), the infiltrative E0771-LG-BM model leads to death mainly via rapid and widespread secondary dissemination and recolonization of secMICs.

### Secondary dissemination tracks in the E0771-LG-BM infiltrative model

As outlined in the introduction, whether metastatic lesions can themselves seed new metastases remains a subject of ongoing debate [6]. Our findings from the E0771-LG-BM model provide strong evidence that secMICs can indeed give rise to new metastatic lesions, at least within the CNS. Both the retrospective histological analysis (Fig. 2) and exploratory prospective MRI imaging study (Fig. 3) revealed secondary lesions located near the meninges, the ventricles and the falx cerebri, suggesting that secMICs do not rely on classical vascular routes. Instead, their locations and progression patterns point toward the use of alternative anatomical dissemination tracks, as previously proposed [6].

**Fig. 4:**
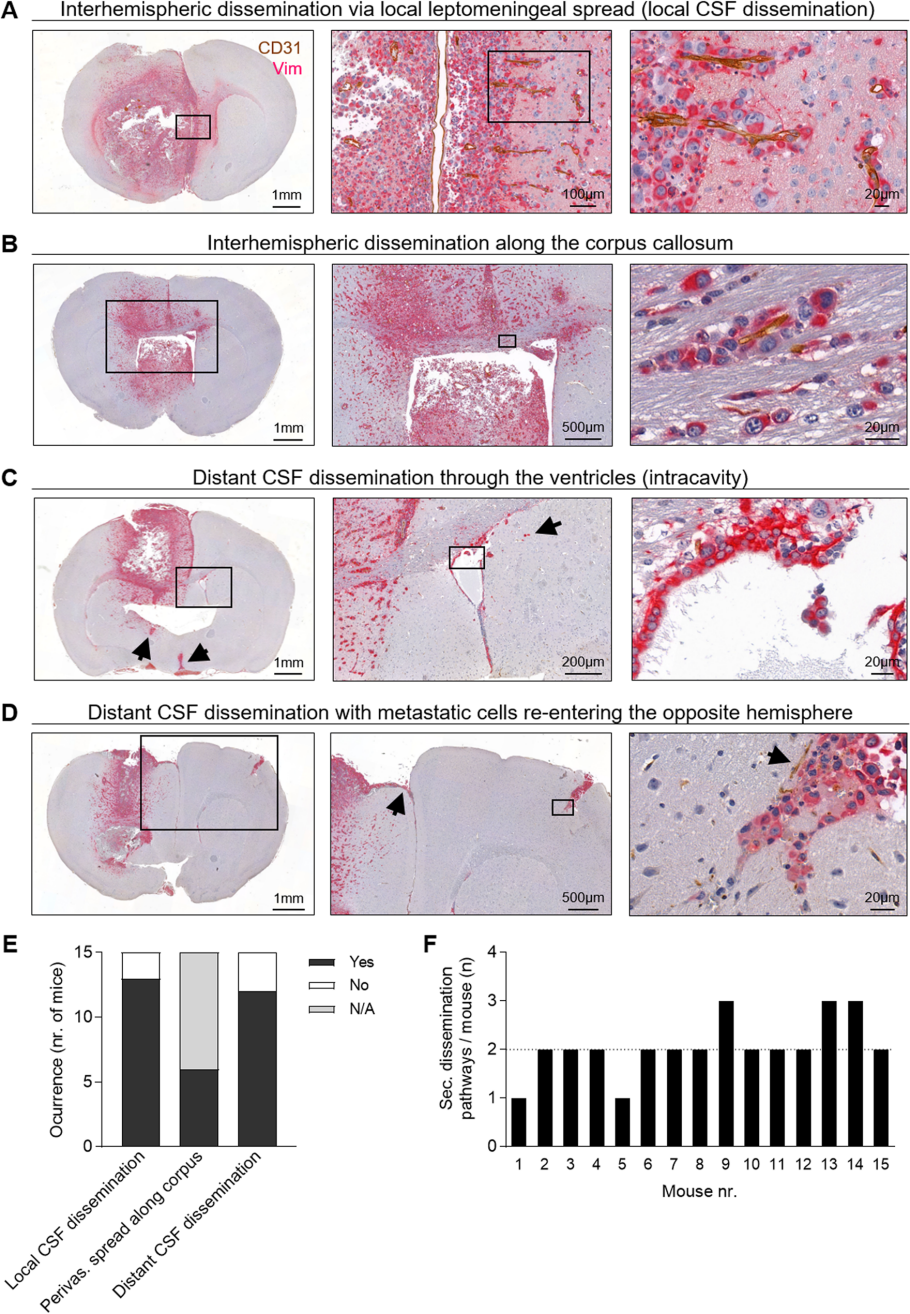
Secondary dissemination tracks of brain metastasis with infiltrative HGPs. **A** Interhemispheric dissemination via local leptomeningeal spread (local CSF dissemination). **B** Perivascular spread along anatomical commissures connecting both hemispheres (e.g., corpus callosum). **C** Distant CSF dissemination through the ventricle system. Arrows indicate the lateral and third ventricles. **D** Distant CSF dissemination with perivascular re-entry of metastatic cells into the contralateral hemisphere. Representative pictures of E0771-LG-BM are shown. Tumor cells and vasculature are double-stained with vimentin (red) and CD31 (brown). **E** Frequency of each secondary dissemination route in E0771-BM (*n* = 15; N/A, not assessable). **F** Co-occurrence of dissemination routes within individual mice. The dashed line indicates the median

To systematically investigate these alternative dissemination pathways, we retrospectively analyzed digital tissue slides from the E0771-LG-BM infiltrative model in detail (*n* = 15). We identified three main anatomical routes through which secMICs disseminate. Firstly, we detected the *interhemispheric dissemination* of metastatic cells to the contralateral hemisphere, occurring via a local leptomeningeal spread (local CSF dissemination, Fig. 4A) or along the corpus callosum (Fig. 4B). In both cases, metastatic cells were observed in nearly every case in association with blood vessels (Fig. 4A-B). Additionally, metastatic cells reached the contralateral hemisphere through the CSF spaces (distant CSF dissemination). In this case, cells entered the CSF compartment via the ipsilateral (right) ventricle, adhered to the ependymal lining, and disseminated within the internal CSF system (Fig. 4C), a process known as *intracavity dissemination* [6]. In some cases, metastatic cells from the external CSF spaces re-entered the contralateral hemisphere, typically along a penetrating blood vessel (Fig. 4D).

Importantly, in all cases we observed that these dissemination pathways were perivascular in nature, with tumor cells tracking along or accumulating adjacent to blood vessels, but not residing within the vascular lumen (Fig. 4A-D). These findings indicate that infiltrative metastatic cells preferentially utilize vessel-associated microenvironments without evidence of true intravascular dissemination, and they support previous hypotheses suggesting that disseminated metastatic cells exploit alternative anatomical routes of secondary dissemination [6].

Quantification of secondary dissemination routes showed that interhemispheric dissemination via local leptomeningeal spread (local CSF dissemination) was the most common pathway (13/15 mice, 86.7%), immediately followed by distant CSF spread (12/15 mice, 80%). Perivascular spread along interhemispheric commissures (e.g. corpus callosum) was present in all mice in which this route could be assessed (6/6, 100%) (Fig. 4E). We observed the co-existence of two or more pathways in 13/15 of the mice, with a median number of two pathways per mouse (Fig. 4F).

Taken together, the three routes identified in this study—local CSF dissemination to contralateral side, perivascular spread using anatomical structures connecting both hemispheres, such as corpus callosum, and distant CSF dissemination with potential secondary intraventricular spread centers—represent distinct anatomical pathways that enable secondary dissemination of the E0771-LG secMICs without engaging the classical vascular routes, as anticipated by Sparrer et al. [6].

### Early onset of HGP formation and secondary dissemination during metastatic colonization

**Fig. 5:**
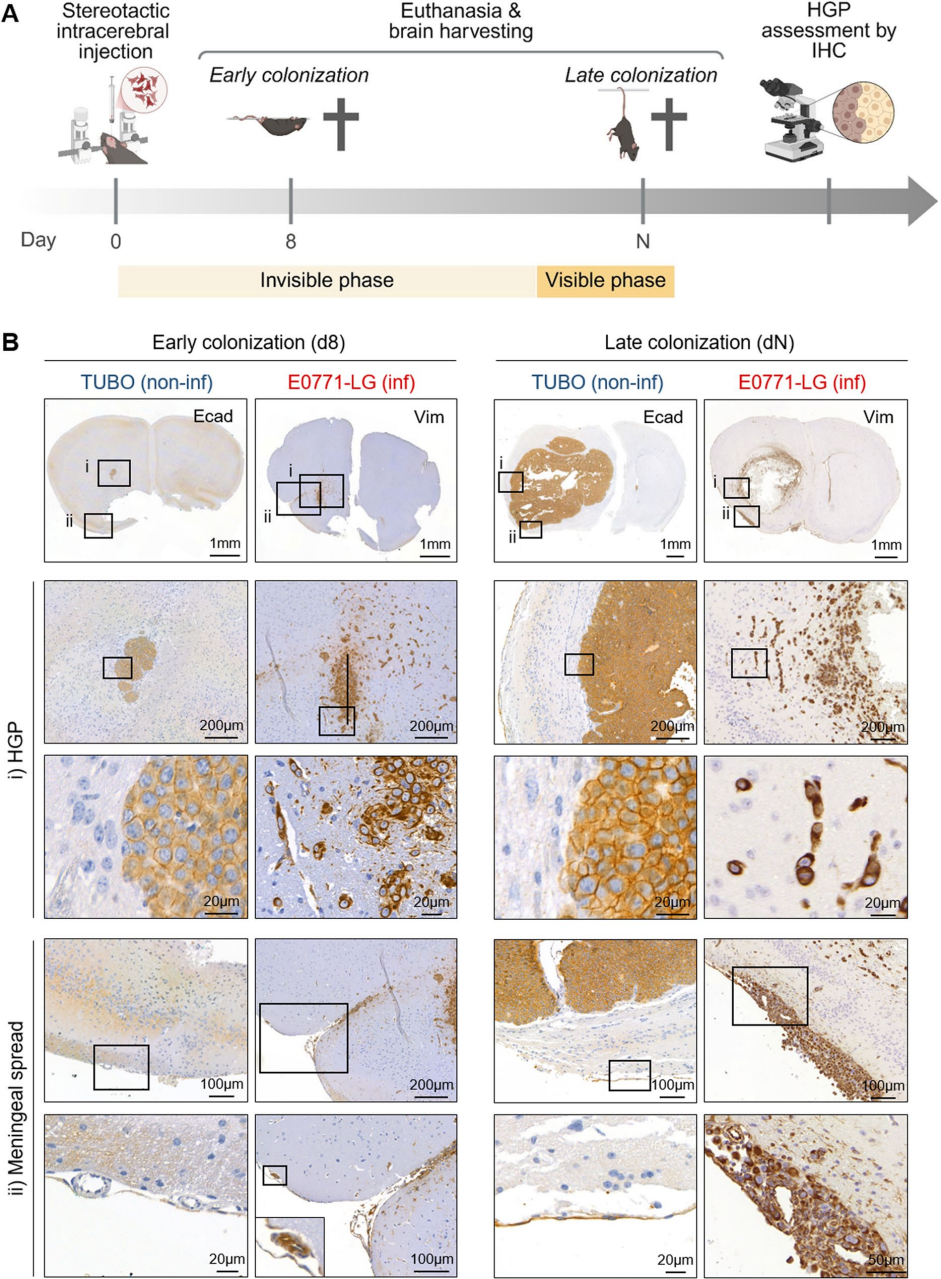
Development of the HGP in BM during metastatic colonization. **A** Schema depicting the experimental setting. Image created with Biorender.com. **B** Representative images of TUBO-BM and E0771-LG-BM depicting the (i) HGP and (ii) meningeal spread during early (left) and late (right) colonization. Tumor cells in tissue sections of TUBO-BM and E0771-LG-BM were stained with E-cadherin (Ecad) or vimentin (Vim), respectively

To investigate the timing of secondary dissemination during metastatic colonization, we referred to our prospective MRI analysis, which showed that the first detectable lesion (M1) appeared around day 14 post-injection—when mice were still asymptomatic (Fig. 3B). We therefore designated day 8 as the ‘early colonization’ time point, representing a clinically silent, “invisible” phase of metastasis. In contrast, ‘late colonization’ corresponded to the onset of neurological symptoms (day N) and the time point when metastatic growth could be visualized by MRI (“visible” phase), marking the humane endpoint and reflecting the presence of overt macro-metastasis (Fig. 5A).

Using immunohistochemistry, we assessed the HGPs at both stages (early and late colonization) in the two models. By day 8, micro-metastases, defined as tumor foci ranging from 0.2 to 2.0 mm in diameter within the brain parenchyma, were already detectable in the ipsilateral hemisphere of both models. Despite the early time point, TUBO-BM and E0771-LG-BM exhibited clearly distinct HGPs: TUBO-BM showed a compact, non-infiltrative HGP, while E0771-LG-BM displayed a loose, infiltrative HGP (Fig. 5B, left panel). Remarkably, these early-stage HGPs closely resembled those observed in macroscopic lesions (Fig. 5B, right panel). Furthermore, in the infiltrative E0771-LG-BM model, secondary dissemination was already evident at this early stage, with secMICs detected along alternative routes such as meningeal spread (Fig. 5B, left panel).

These findings demonstrate that both the HGP and the process of secondary dissemination are established early during metastatic colonization. Contrary to previous assumptions [10], the HGP is not a late-stage adaptation but is imprinted early and persists through the course of metastatic progression.

## Discussion

Neurological death accounts for approximately 15–50% of deaths in patients with brain metastases [23–25]. However, the specific mechanisms leading to CNS failure remain poorly understood. In this study, we present two distinct models of breast cancer brain metastasis, each illustrating different pathophysiological mechanisms leading to neurological death reflected by their HGPs.

On the one hand, the non-infiltrative TUBO-BM model remained confined to the ipsilateral hemisphere, causing local mass effect and displacement of adjacent brain tissue. This expansion resulted in enlargement of the right hemisphere at the expense of the left, producing a marked midline shift in accordance with the Monro-Kellie doctrine [7]. Moreover, the non-infiltrative model revealed multiple layers of reactive astrocytes surrounding and containing the single metastatic lesion. These results demonstrate that the progressive expansion of a single, localized metastasis is the pathophysiological driver of CNS failure and, ultimately, neurological death in brain metastases with non-infiltrative HGPs. On the other hand, in the diffuse infiltrative E0771-LG-BM model, tumor cells spread beyond the initial lesion (M1) to seed additional metastases (M2, and occasionally M3) at distant sites within the brain parenchyma, leading to a widespread destruction of the organ and contributing to neurological decline and death. We cannot rule out a contribution of midline shift to neurological decline in the infiltrative E0771-LG-BM model, as 5/14 of mice exhibited a midline shift in the retrospective analysis. However, this was not accompanied by a significant increase in ipsilateral hemisphere volume, as observed in the non-infiltrative model, suggesting only a marginal effect.

To our knowledge, this is the first study providing direct evidence of secondary dissemination and successful recolonization of the contralateral brain hemisphere. Given the experimental setup—where tumor cells are injected directly into the brain—these secondary lesions could not have originated from primary tumor–derived metastasis-initiating cells (primMICs), as previously proposed [14], but instead must arise from pre-existing cerebral metastatic lesions (secMICs). Importantly, we demonstrate that secondary metastases (M2-M3, derived from secMICs) grow significantly faster than the initial lesion (M1). These results echo earlier recolonization experiments, where reinjection of cells that have already successfully colonized a target organ led to a faster onset of further metastatic lesions [26], and highlight the high efficiency of secondary spread.

Notably, secMICs appear to exploit distinct routes to reach distant brain regions, bypassing traditional vascular dissemination pathways, as previously described [6]. Our results show that metastatic cells disseminate to the contralateral hemisphere either through local leptomeningeal spread or along pre-existing anatomical structures connecting both hemispheres, such as the corpus callosum. In the infiltrative E0771-LG-BM model, tumor cells from the M1 can also enter the cerebrospinal fluid (CSF), spread along CSF spaces, and give rise to secondary metastases (M2) in both the brain parenchyma and meninges (distant CSF spread). Perivascular spread is frequently observed along these structures. However, in the case of the corpus callosum-mediated dissemination, a purely perivascular dissemination is anatomically unlikely, as the vascular supply is largely ipsilateral. Tumor cells within the corpus callosum are also found without direct association with blood vessels, suggesting that migration along axonal tracts is a plausible mechanism. Moreover, we cannot definitively exclude that some cells may reach the contralateral hemisphere retrogradely via the CSF along subependymal vessels and subsequently spread through the brain tissue. Acknowledging these limitations, our observations reveal at least three alternative dissemination routes for brain metastasis in this model, underscoring a previously underappreciated complexity in which secondary lesions emerge through spatially and mechanistically diverse pathways, similar to patterns described in glioblastoma [27].

Meningeal involvement is recognized as a strong independent predictor of neurological death in brain metastasis, especially in patients with EGFR-mutant NSCLC [23]. Remarkably, we recently described a potential link between infiltrative HGPs and the development of meningeal metastases in the MetInfilt trial, a prospective study specifically designed to collect samples from the MMPI_brain_ and evaluate the HGP of brain metastases [13]. This observation is consistent with that of Dankner et al., who reported a significant correlation between the degree of infiltration in brain metastases and the risk of meningeal spread [12]. The present study corroborates the feasibility of these findings and highlights the HGP as a potential surrogate morphological marker for meningeal spread in infiltrative metastasis.

Our findings indicate that the HGP is intrinsic to tumor cells, as previously pointed out by others [12, 28]. This also aligns with previous observations in liver metastases, where the HGP was described as an epigenetically driven biological event [29]. Furthermore, we demonstrate that the HGP is established early, during the clinically undetectable phase of metastatic colonization, consistent with findings reported for glioblastoma [28]. Recent studies have also identified two distinct HGPs—perivascular and spheroidal—during early metastatic colonization [30]. Collectively, these findings support the notion that the intrinsic program governing the HGP is initiated in parallel with the colonization of the host organ.

Moreover, features associated with neurological decline—such as local metastatic expansion in non-infiltrative models and secondary dissemination in infiltrative ones—emerge timely in the disease course, likely beginning soon during colonization of the initial lesion. This observation may have important consequences for the clinical management of patients with brain metastases, particularly in guiding the choice and sequencing of local and systemic therapies. For instance, a non-infiltrative HGP may predict elevated ICP and warrant early intervention with steroids, decompressive surgery or radiosurgery. Conversely, patients with infiltrative metastases—who are at risk for secondary dissemination—may benefit more from systemic therapy, and surgical intervention should be minimized to reduce iatrogenic spread, or combined with neoadjuvant radiotherapy. Given the emerging ability to non-invasively identify infiltrative HRI [GPs via M 13, 31] and the consistency of HGPs across multiple lesions within individual patients [11, 32], these findings underscore the potential of the HGP as a histological marker to inform and guide future clinical decisions. These findings suggest that incorporating HGP assessment into future pathological reports for brain metastases may be beneficial.

The primary limitation of our study lies in the method used to induce CNS metastasis. Because tumor cells were directly implanted into the brain, we cannot fully exclude the possibility that secondary dissemination may be a result of iatrogenic factors. However, since orthotopic implantation was employed in both models, it is noteworthy that only the E0771-LG-BM model exhibited a higher capacity of intra-organ dissemination and recolonization by secMICs. Nevertheless, complementary validation of our findings using intracardiac or intracarotid injection models, which include additional steps of the metastatic cascade such us the seeding and extravasation of tumor cells into the CNS, would contribute to strengthen the generalizability and translational relevance of our conclusions.

## Conclusions

In conclusion, this study identifies distinct mechanisms leading to neurological decline in brain metastases and demonstrates that the underlying cause of death is closely tied to the HGP. Our results challenge the traditional “five-step” vascular-centric model of metastasis by providing preclinical evidence that intra-organ secondary dissemination is biologically possible and showing that different dissemination paths (co-)exist in this process. These insights have potential to reshape clinical strategies for managing brain metastases. Incorporating HGP assessment into routine pathological reporting could support clinical decision-making and improve patient outcomes across various stages of care.

## Supplementary Information

Supplementary Material 1.

## Data Availability

The datasets used and/or analyzed during the current study are available from the corresponding author on reasonable request.

## Abbreviations

BCBM: Breast cancer brain metastasis
CNS: Central nervous system
CSF: Cerebrospinal fluid
CI: Colonization Index
CTL: Control
DMEM: Dulbecco’s Modified Eagle’s Medium
FCS: Fetal calf serum
HGP: Histological growth pattern
IHC: Immunohistochemistry
ICP: Intracranial pressure
LH: Left hemisphere
MMPI_brain_: Macro-metastasis–brain parenchyma interface
MRI: Magnetic resonance imaging
NSCLC: Non-small cell lung cancer
OS: Overall survival
primMICs: Primary metastasis-initiating cells
RH: Right hemisphere
secMICs: Secondary metastasis-initiating cells
ZTL: *Zentrale Tierlaboratorien* (*engl*. Central Animal Facility)
