# Supplementary material for: Local metastatic expansion versus secondary intra-organ dissemination: two causes of neurological death explained by fundamentally different metastatic colonization patterns
Source: Mol Cancer. 2026 Jan 24;25:17. doi: 10.1186/s12943-026-02574-0 (PMC12849154; doi:10.1186/s12943-026-02574-0)
Supplement: Supplementary file 1 — Supplementary Material 1. [file 12943_2026_2574_MOESM1_ESM.docx]

**Supplementary Figures**

**
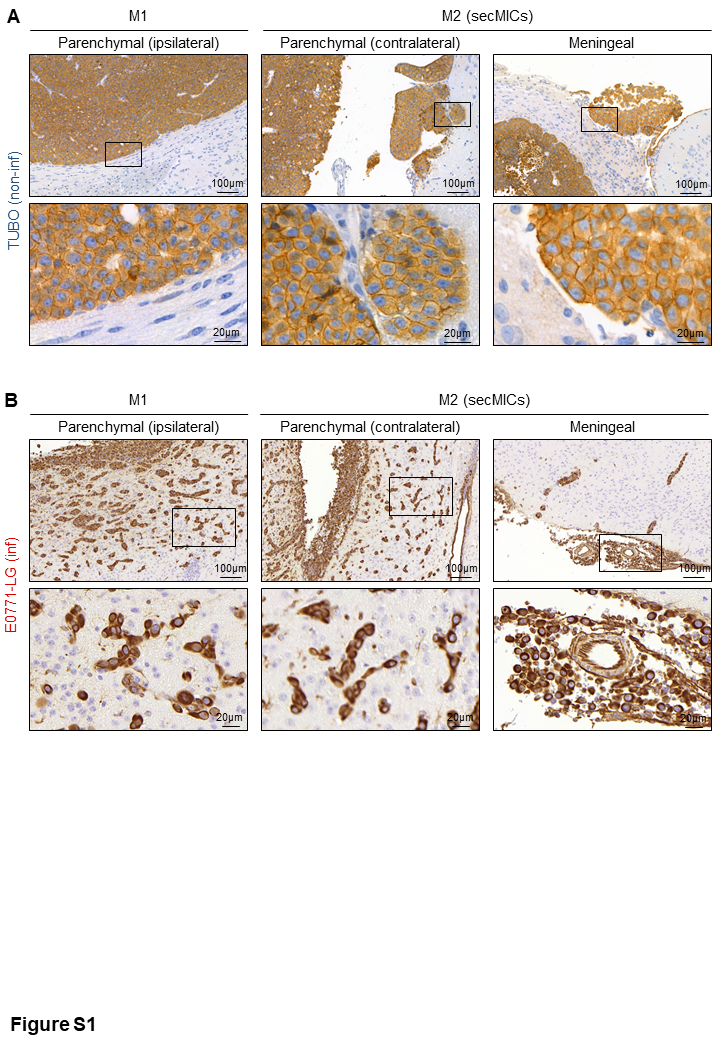
**

**Figure S1: Evaluation of HGP in primMICs and secMICs in experimental models of brain metastasis with infiltrative vs. non-infiltrative HGPs.** HGP of primMICs (parenchymal metastasis at the ipsilateral site) and secMICs (parenchymal metastasis at the contralateral site and meningeal growth) in **(A)** TUBO-BM and **(B)** E0771-LG-BM. Representative pictures are shown. Tumor cells in tissue sections of TUBO-BM and E0771-LG-BM were stained with E-cadherin (Ecad) or vimentin (Vim), respectively.

**
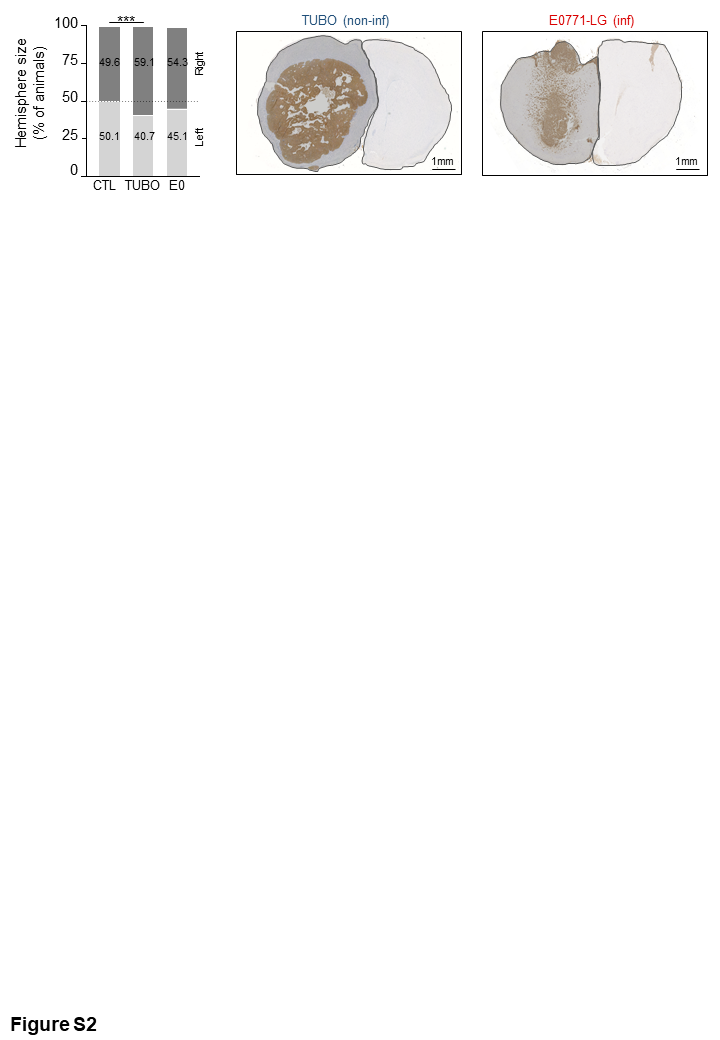
**

**Figure S2: Retrospective histological evaluation of tumor growth in experimental models of brain metastasis with infiltrative vs. non-infiltrative HGPs.** Quantification of hemisphere size in control mice (CTL), TUBO-BM and E0771-LG-BM. Representative pictures are shown. One-way ANOVA followed by Tukey's multiple comparisons test (*P* < 0.001).

**
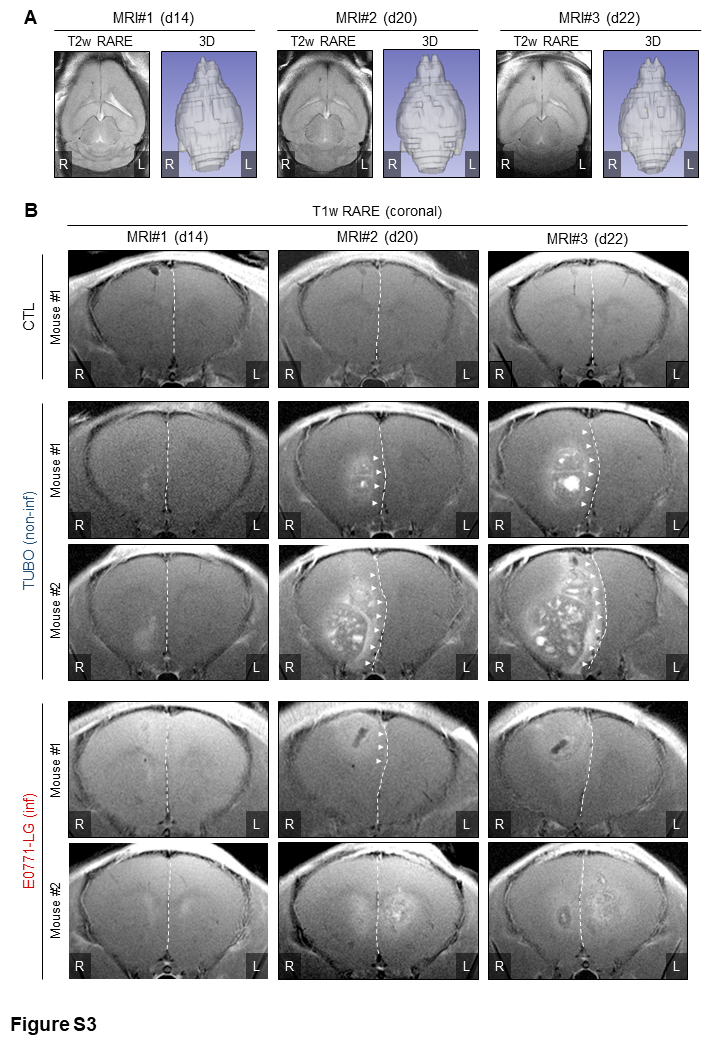
**

**Figure S3: Prospective MRI evaluation of growth dynamics in experimental models of brain metastasis with infiltrative vs. non-infiltrative HGPs. (A)** Horizontal T2-weighted MR images and 3D reconstructions of control mice (CTL) at day 14 (MRI#1), 20 (MRI#2) and 22 (MRI#3) after stereotactic injection. **(B)** Coronal T1-weighted MR images of mouse brains of control (CTL), TUBO-BM and E0771-LG-BM at day 14 (MRI#1), 20 (MRI#2) and 22 (MRI#3) after tumor cell inoculation. The arrows indicate the metastatic lesions. The dashed lines show the brain midline.
